# SurvNet Electronic Surveillance System for Infectious Disease Outbreaks, Germany

**DOI:** 10.3201/eid1310.070253

**Published:** 2007-10

**Authors:** Gérard Krause, Doris Altmann, Daniel Faensen, Klaudia Porten, Justus Benzler, Thomas Pfoch, Andrea Ammon, Michael H. Kramer, Hermann Claus

**Affiliations:** * Robert Koch Institute, Berlin, Germany; †Picoware GmbH, Berlin, Germany; ‡ Ministry of Health, Berlin, Germany; 1 Current affiliation: Epicentre, Geneva, Switzerland; 2 Current affiliation: European Centre for Disease Prevention and Control, Stockholm, Sweden

**Keywords:** Surveillance, emerging infectious diseases, outbreaks, notifiable diseases, international health regulations, research

## Abstract

In 2001, the Robert Koch Institute (RKI) implemented a new electronic surveillance system (SurvNet) for infectious disease outbreaks in Germany. SurvNet has captured 30,578 outbreak reports in 2001–2005. The size of the outbreaks ranged from 2 to 527 cases. For outbreaks reported in 2002–2005, the median duration from notification of the first case to the local health department until receipt of the outbreak report at RKI was 7 days. Median outbreak duration ranged from 1 day (caused by *Campylobacter*) up to 73 days (caused by *Mycobacterium tuberculosis*). The most common settings among the 10,008 entries for 9,946 outbreaks in 2004 and 2005 were households (5,262; 53%), nursing homes (1,218; 12%), and hospitals (1,248; 12%). SurvNet may be a useful tool for other outbreak surveillance systems because it minimizes the workload of local health departments and captures outbreaks even when causative pathogens have not yet been identified.

## Abstract 2

Electronic Surveillance System for Infectious Disease Outbreaks, Germany

This system has managed detailed information on 30,578 disease outbreaks.

Surveillance of infectious disease outbreaks is important because outbreaks often require immediate intervention by the public health service. In addition, outbreaks may indicate deficiencies in infection control management and provide unique opportunities to investigate clinical and epidemiologic characteristics of the infectious agents, particularly in emerging infectious diseases. Timely and comprehensive outbreak reports need to be available not only at the affected administrative level but also at state, national, and international levels to detect and control multistate outbreaks (*1**–**4*). Electronic documentation and transmission of data are needed for rapid information exchange between institutions in charge of conducting, coordinating, or reporting control measures and should minimize additional work load for the public health service (*5*).

International regulations have resulted in increased requirements for outbreak reporting from the local to the international level (*6**,**7*). One of the major changes in the new International Health Regulations enacted in May 2005 is that infectious disease outbreaks of international concern must be reported to the World Health Organization, irrespective of the pathogens involved (*8*). Moreover, member states of the European Union are already obligated to report foodborne outbreaks to the relevant European Union institution according to the regulation on monitoring of zoonoses and zoonotic agents (*9*).

Outbreak surveillance for emerging infectious diseases is a particular challenge because small independent outbreaks may occur before they are recognized as part of a larger epidemiologic phenomenon. The complexity, the prolonged persistence of outbreaks, and the differing degree to which outbreaks are investigated locally make it much more difficult to ensure standardized and timely surveillance of outbreaks compared with surveillance of sporadic cases (*10*). To overcome these problems, the RKI (the federal institution responsible for infectious disease surveillance in Germany) developed the software and implemented an electronic outbreak reporting system (SurvNet) as part of its existing electronic surveillance system for notifiable diseases. SurvNet was fully implemented in January 2001 at all administrative levels of the German Public Health system and, in January 2006, at all levels of the German armed forces. The objective of the system is timely and easily retrievable epidemiologic information exchange on outbreaks at the local, state, and national levels. We describe the system, present epidemiologic aspects of reported outbreaks, and discuss the strengths and weaknesses after 5 years of practical use in Germany.

## Material and Methods

### Electronic Transmission of Data

All 431 local health departments in Germany verify locally identified notifiable diseases with reference to national case definitions and send case reports electronically through the 16 state health departments to the national surveillance unit at RKI. The SurvNet software organizes the electronic transmission of case-based datasets from peripheral databases in each local health department to databases of the respective state health department and finally to the RKI (*11**,**12*). The system transmits data to the RKI on all cases in Germany but without identifiable information on the persons involved. In contrast, a local health department has full data on all cases from their jurisdiction (*11*). The data collected in this system includes demographic characteristics, time, place, diagnostics, case definition criteria, exposure to risk factors, and associations with outbreaks as well as administrative data on where, when, and by whom the dataset is being installed and modified.

### Outbreak Reporting

Single case records can be linked together in the SurvNet database by creating an outbreak report as a new database unit. Several outbreak reports at the local level can be further combined, which results in meta-outbreak reports (Figure). This so called “inverted tree” structure allows documentation of multicounty and multistate outbreaks (*13*). Outbreak reports can also be linked with outbreaks that initially were thought to be unlinked but are later identified as being part of the same epidemiologic event. Staff at local or state health departments, as well as at RKI, can electronically link outbreaks on the basis of epidemiologic evidence such as person, place, time, and pathogen; they can also manually enter descriptive categorizations based on the information provided by the outbreak reports that form part of this meta-outbreak (Appendix Figure).

**Figure:**
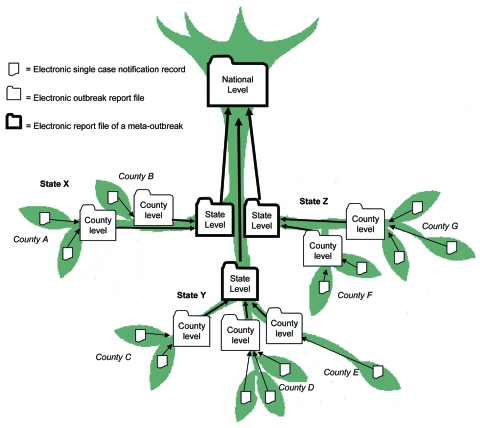
Inverted tree structure for organizing electronic outbreak reporting at different administrative levels.

### Information Structure

The qualitative characteristics of the outbreak are covered by 7 sections: geographic setting, food consumption, bloodborne diseases, animal contact, waterborne diseases, person-to-person contact, and molecular fingerprinting. Each section includes a list of standardized items, of which >1 can be selected. Food consumption, for example, contains a selection of standardized food items defined by the first hierarchical order of the Eurocode 2 Food Coding System (*14*), a system developed to serve as a standard instrument for nutritional surveys in Europe.

Each selected exposure in the different sections is additionally categorized by using standardized evidence categories (Table 1). These range from “exposure confirmed by significant association in case-control or cohort study” to “majority of the cases of this outbreak had this particular exposure.” For example, the category “breach of applicable standard recommendations supports epidemiologic link” is applicable if an outbreak investigation associated with meat consumption shows that consumed meat was not properly cooked. In addition to these standardized variables, results from molecular analysis of pathogens and complementary narrative information, such as anecdotal evidence, can also be included and categorized as “other information.”

**Table 1:** Levels of evidence supporting associations with named exposures, reported infectious disease outbreaks, 2004 and 2005, Germany

Level of evidence	No. exposures (%)						
	Geographic setting	Person-to-person	Food	Blood	Water	Animal	All
Pathogen (in linked person or environmental sample) indicates epidemiologic link	98 (1.4)	3,544 (64.7)	65 (3.8)	4 (1.3)	3 (3.5)	8 (13.6)	3,722 (25.2)
Significant association by epidemiologic study (e.g., case control or cohort study)	763 (10.7)	369 (6.7)	139 (8.2)	148 (47.3)	5 (5.9)	2 (3.4)	1,426 (9.7)
Most cases had same exposure	6,063 (85.2)	1,361 (24.8)	1,342 (79.4)	60 (19.2)	44 (51.8)	32 (54.2)	8,902 (60.4)
Breach of applicable standard recommendations supports epidemiologic link	N/A	N/A	57 (3.4)	28 (8.9)	3 (3.5)	2 (3.4)	90 (0.6)
Other reasons	191 (2.7)	207 (3.8)	88 (5.2)	73 (23.3)	30 (35.3)	15 (25.4)	604 (4.1)
All entries	7,115 (100)	5,481 (100)	1,691 (100)	313 (100)	85 (100)	59 (100)	14,744 (100)
All outbreaks	7,074	5,400	1,637	311	85	59	14,566

Outbreaks are generally linked to >1 pathogens identified as the causative agent(s) of the outbreak. For notifiable pathogens, the system provides a specific set of variables to allow validation against the respective case definition. Regarding pathogens for which sporadic cases are not notifiable, the agent can be selected from a list of 133 known human pathogens. If a pathogen cannot be detected, the cases can still be transmitted as part of an outbreak. The system automatically generates an outbreak profile consisting of general tables and graphs on the descriptive epidemiology of the outbreak, including epidemic curves, categorizations by age and sex, and geographic distribution of cases. Statistical overviews of reported outbreaks are published in the annual epidemiologic report on infectious diseases in April or May after the reporting year (*15*). Outbreaks of special interest are highlighted in short profiles in the weekly Epidemiological Bulletin, which may be followed by full outbreak reports in the same bulletin or other scientific journals (*16*).

### Outbreaks and Statistical Analyses

Data presented in this article cover all outbreaks reported to the RKI from 2001 through December 2005 as of July 31, 2006. After the German infectious disease control law was passed, an outbreak was defined as >2 cases with an epidemiologic link (*12*). A case was considered epidemiologically confirmed if the clinical picture and an epidemiologic link to at least 1 laboratory-confirmed case was present as specified by the national case definition for the respective disease (*17*), e.g., a person with diarrhea and no laboratory diagnosis who had ingested the same implicated food item as >1 patients with laboratory confirmed salmonellosis. Outbreaks, which were part of a meta-outbreak, were not counted separately because the case data were already included in the respective meta-outbreak (Figure).

Unless otherwise specified, analyses were limited to outbreaks caused by notifiable pathogens defined by the national surveillance case definitions (*18*). Duration of an outbreak was defined as the interval between the onset of the first and the last case of the outbreak. Date of diagnosis was used if date of onset was missing. Reporting delay between the different public health levels was based on the electronic time stamps for entering respective data into the database and arrival at the RKI. Reporting delay was computed for the years 2002 through 2005, as technical constraints did not allow these analyses for the 2001 data. Chi-square testing was used to compare the proportion of outbreaks with food as a source for various pathogens.

The system for documenting qualitative descriptions of the outbreaks has undergone major revisions over the years. Therefore, data on these details are presented only for the years 2004 and 2005, to ensure a consistent and comparable data structure. Microsoft SQL Server 2005 (Microsoft Corp., Redmond, WA, USA) was used for database management. For the descriptive statistics, we used Statistical Package for the Social Sciences (SPSS) 15.0 for Windows, Version 15.0/1 (SPSS Inc., Chicago, IL, USA).

## Results

From January 2001 through December 2005, a total of 30,578 outbreaks associated with notifiable pathogens were reported to RKI. Of 1,340,487 cases of notifiable diseases reported to RKI during this period, 253,720 cases (19%) were part of a reported outbreak; the rest were reported as sporadic cases (Table 2). Of these outbreaks, 90% were caused by pathogens of the intestinal tract (e.g., *Salmonella*, norovirus, rotavirus, hepatitis A virus, enteropathogenic *Escherichia coli*, and *Campylobacter*), and 10% (3,201) were caused by influenza virus (713), *Mycobacterium tuberculosis* (637), measles virus (501), and others (1,350, by 47 notifiable pathogens) (*12*). The size of the outbreaks ranged from 2 to 527 cases. Table 3 shows the number and duration of outbreaks by size and pathogens and indicates that duration increases with the size of the outbreak. The longest median durations were observed in outbreaks caused by hepatitis A virus (22 days) and by *M. tuberculosis* (73 days).

**Table 2:** Total number of cases and outbreaks of notifiable disease, 2001–2005, Germany

Data point	2001	2002	2003	2004	2005	All
Total no. reported cases	245,133	284,425	252,119	267,130	291,680	1,340,487
No. (%) cases as part of reported outbreaks	22,146 (9.0)	67,498 (23.7)	48,855 (19.4)	58,204 (21.8)	57,017 (19.5)	253,720 (18.9)
No. outbreaks (any size)	3,981	6,914	6,261	6,340	7,082	30,578
No. (%) outbreaks with <5 cases	3,118 (78.3)	4,573 (66.1)	4,524 (72.3)	4,007 (63.2)	4,945 (69.8)	21,167 (69.2)
No. (%) outbreaks with >5 cases	863 (21.7)	2,341 (33.9)	1,737 (27.7)	2,333 (36.8)	2,137 (30.2)	9,411 (30.8)

**Table 3:** Size and median duration* of outbreaks by pathogen, 2001–2005, Germany

Outbreak type, no. cases/median duration	Size of outbreak								
	2	3	4	5	6–9	10–49	50–99	>100	All
*Salmonella*	5,855/2	2,134/2	1,006/2	504/2	721/3	636/6	34/16	10/22.5	10,900/2
Norovirus	1,169/2	641/3	469/3	342/4	984/6	3,694/9	636/17	140/26	8,075/7
Rotavirus	2,570/3	941/4	425/6	176/7	396/8	532/13	28/29	2/49	5,070/4
*Campylobacter*	2,032/1	390/2	123/3	44/3	45/4	34/8	1/8	0	2,669/1
*Mycobacterium tuberculosis*	454/62.5	102/105	44/103	16/229.5	19/282	2/253.5	0	0	637/73
Influenza	420/2	163/3	67/3	19/6	17/5	25/15	2/38.5	0	713/3
Hepatitis A virus	227/18	102/22	42/23	33/44.5	32/51.5	7/106	1/77	1/106	445/22
*Giardia*	192/2.5	52/12.5	27/13	4/12	3/74	4/27	0	0	282/4
*Salmonella paratyphi*	13/2	3/20	0	0	0	1/40	0	0	17/4
Other	1,052/2	318/6	134/8	61/14	88/17	102/40	8/83.5	7/123	1,770/6
All pathogens	13,984/2	4,846/3	2,337/4	1,199/4	2,305/6	5,037/9	710/18	160/27	30,578/3

*Duration and median duration are given in full days.

In addition to the 30,578 outbreaks associated with notifiable pathogens, 772 outbreaks were reported but not associated with any specific pathogen; 155 outbreaks were associated with pathogens that are not notifiable as single cases. Among these 155 outbreaks, 25 (16.8%) were associated with varicella-zoster virus, 26 (16.1%) *Staphylococcus* spp., 24 (15.5%) *Sarcoptes scabiei*, 16 (10.3%) coxsackie virus, 15 (9.7%) adenovirus (nonconjunctivitis), 11 (7.1%) *Streptococcus* spp., 10 (6.5%) astrovirus, and 28 (18.1%) outbreaks with 1 of 16 other pathogens. The distribution of these pathogens did not show any significant change over the years. The size, duration, and reporting delay for these different kinds of outbreaks are compared in Table 4.

**Table 4:** Comparison among outbreaks, 2001–2005, Germany*

Characteristic	Linked to notifiable pathogens	Linked to nonnotifiable pathogens	No link to any specific pathogen
No. outbreaks	30,578	155	772
Median duration, d	3	9	4
Median no. cases per outbreak (minimum, maximum)	3 (2,527)	8 (2,153)	10 (2,110)
Median duration from report of first case until outbreak report filed at local health department, d	26,597	154	772
Median duration between filing of outbreak report at local health department until arrival of report at RKI, d	1	0	0
Median duration from report of first case until outbreak report arrives at RKI, d	2	2	3

*RKI, Robert Koch Institute.

A location setting was reported for 9,946 outbreaks (33%). Of 10,008 listed items, the most frequently named categories were households (5,262; 53%), nursing homes (1,218; 12%), hospitals (1,248; 12%), and kindergartens (783; 8%) (Table 5).

**Table 5:** Locations of outbreaks by pathogen, 2004 and 2005, Germany*

Location	No. (%)						
	Norovirus (n = 3,141)	*Salmonella* spp. (n = 2,703)	Rotavirus (n = 1,985)	*Campylobacter* spp. (n = 1,005)	Hepatitis A (n = 139)	Others (n = 973)	All (n = 9,946)
Household	395 (13)	1,993 (73)	1,338 (67)	758 (75)	102 (72)	676 (69)	5,262 (53)
Nursing home	1,040 (33)	24 (1)	136 (7)	5 (0)	0 (0)	13 (1)	1,218 (12)
Kindergarten	368 (12)	61 (2)	290 (15)	7 (1)	9 (6)	48 (5)	783 (8)
Hospital, laboratory	1,035 (33)	20 (1)	175 (9)	5 (0)	1 (1)	12 (1)	1,248 (12)
Hotel, cruise ship	58 (2)	169 (6)	16 (1)	120 (12)	12 (8)	93 (9)	468 (5)
Restaurant	72 (2)	258 (9)	1 (0)	48 (5)	3 (2)	10 (1)	392 (4)
Other location	34 (1)	80 (3)	28 (1)	36 (4)	7 (5)	61 (6)	246 (2)
School, university	34 (1)	18 (1)	0	7 (1)	8 (6)	20 (2)	87 (1)
Special event, festival, etc.	24 (1)	55 (2)	4 (0)	9 (1)	0	6 (1)	98 (1)
Work place	37 (1)	21 (1)	0	10 (1)	0	17 (2)	85 (1)
Dormitory, military casern	56 (2)	9 (0)	9 (0)	1 (0)	0	11 (1)	86 (1)
Bus/ train, etc.	2 (0)	5 (0)	0	4 (0)	0	2 (0)	13 (0)
Prison	2 (0)	4 (0)	3 (0)	0	0	3 (0)	12 (0)
Refugee camp	0	2 (0)	0	0	0	8 (1)	10 (0)
Total number of listed items	3,157 (100)	2,719 (100)	2,000 (100)	1,010 (100)	142 (100)	980 (100)	10,008 (100)

*Outbreaks may be reported in >1 location.

In the 13,422 outbreaks reported in 2004 and 2005, at least 1 exposure associated with the outbreak was reported in 10,205 (76%) outbreaks, which added up to a total of 22,001 field entries (average 2.2 entries per outbreak). For 15,978 (66%) of these 24,208 field entries, an evidence category was provided by the reporting local health departments. The distribution of these categories is shown in Table 1. In 954 (9.3%) of all 10,205 outbreaks linked to a specific exposure, the evidence of this linkage was based on a statistically significant association in a case-control or cohort study. For the 2,195 outbreaks with >10 cases, this type of evidence was reported in 248 (11.3%) outbreaks, compared with 706 (8.8%) of the 7,998 outbreaks with <10 cases relative risk (RR) = 1.3, χ^2^ = 12.5, p<0.001.

For 1,637 (64%) of the 2,554 outbreaks in 2004 and 2005 that were linked to food, information was available about the evidence on which the association was based. For 204 (12%) of these, the linkage was supported by either statistically significant association or by detection of the causative pathogen in a food sample. In these 12% of outbreaks in which the exposure linkage was supported by the 2 latter methods, the proportion of outbreaks linked to food varied between causative pathogens. In 999 of such outbreaks caused by *S.* *enteritidis* spp., 14% (141) were associated with food either by a statistically significant association or by detection of the causative pathogen in a food sample; this association was found for 8% (28 of 359) *Campylobacter* outbreaks, 1% (16 of 1,239) norovirus outbreaks, and 0.2% (2 of 940) rotavirus outbreaks (χ^2^ = 215.6, p<0.001).

The median delay from receipt of the first case notification until electronic filing of an outbreak report at the local health department was 4 days in 2002, 1 day in 2003, and 0 (i.e., same day) in 2004 and 2005. The median reporting delay from electronic filing of the outbreak report at the local health department to arrival of the electronic report at RKI was 1 day in 2002, 2 days in 2003, and 3 days in 2004 and 2005. The overall median delay from receipt of the first case notification by the local health department until arrival of the electronic outbreak report at RKI remained stable at 7 days from 2002 through 2005.

## Discussion

Effective surveillance of emerging infectious diseases requires a system able to transmit locally detected outbreak reports at an early stage, for example, when an epidemiologic investigation is still under way. The SurvNet outbreak surveillance system ensures continuous updating of the outbreak reports as more cases are identified or linked to the outbreak, long before an outbreak investigation has been finalized in a written report. This system also facilitates rapid electronic linkage of apparently independent outbreaks, for example, in different states, enabling subsequent analysis of the entire meta-outbreak. Although legally not considered an outbreak, single case notifications of rare diseases with strong public health implications (e.g., anthrax) will of course be captured through the SurvNet system as single case records and will result in immediate investigation and action by local authorities.

During the past 5 years, the SurvNet outbreak surveillance system has managed standardized collection, transmission, and reporting of complex information generated by outbreak investigations of all 431 local health departments in Germany. As shown in this report, the system also covers diseases for which the causative pathogen is either not identifiable or identified but not notifiable when occurring sporadically. Local health departments become aware of such incidents because outbreaks or infections with new or unknown pathogens that are potentially dangerous to the public are also notifiable under German law. This ability makes SurvNet particularly useful for the surveillance of emerging infectious diseases for which laboratory diagnosis may often be delayed or not yet possible. SurvNet has the advantage of managing epidemiologic information that laboratory-based systems or syndromic surveillance systems alone cannot easily provide. Essential epidemiologic evidence can be retrieved only through local outbreak investigations that are usually conducted by local health departments (*19*), which constitute the most critical component of outbreak detection and investigation. (*3**,**5*).

The SurvNet system appears to capture far more outbreaks per population than published collections of outbreak reports in other countries. For example, the Electronic Foodborne Outbreak Reporting System managed by the Centers for Disease Control and Prevention (CDC) has listed 1,319 foodborne outbreaks in the year 2004 within the United States (estimated incidence rate of 0.4 outbreaks per 100,000) compared with 1,263 foodborne outbreaks captured in SurvNet in that same year in Germany (incidence rate 1.5/100,000) (*20*). A similar difference is seen when comparing data from the SurvNet system in Germany with surveillance data on foodborne outbreaks in England and Wales or to the number of *Salmonella* outbreaks collected by different surveillance systems in France (*21**–**23*). These differences could be due to different case definitions, true difference in incidence caused by significantly poorer food safety in Germany, or other reasons. However, the higher outbreak incidence rate in the SurvNet system is likely the result of its higher sensitivity, at least in part. Technically, the system is an integral part of the routine surveillance for notifiable diseases, which means that local health departments are required to enter and administer only outbreak-related data, because most of the information from the database of notifiable disease cases is being used in both systems. This synergism is likely to encourage local health departments to use the system and thus improve its sensitivity. Because all of the outbreaks identified in this system are events identified and investigated by local public health staff, the positive predictive value of detecting a true outbreak is likely to remain high. This is one of the major advantages of SurvNet compared with outbreak detection systems based on statistical algorithms of case reports. Data from SurvNet may, in fact, serve as the standard to validate statistical outbreak detection algorithms (*24*). SurvNet may also provide data to identify prognostic criteria that would help in forecasting the natural development of a specific outbreak (*25**–**28*). However, details of outbreak reports have not yet been systematically validated, so careful interpretation of the information is essential. Compared with CDC’s outbreak reports analyzed by Ashford and colleagues, the SurvNet system appears to be much timelier, although a direct comparison is not possible because the types of investigated outbreaks and the definitions of the reporting delays are not directly comparable (*5*).

The European Food and Safety Authority is currently building a reporting system for foodborne outbreaks in the European Union using the methods developed in SurvNet (pers. comm., P.Mäkelä, European Food Safety Authority). The Eurocode 2 System used here to categorize food appears to be user friendly and is available on the Internet with instructions on how to categorize food items that appear difficult to assign to 1 category (*14*). Most outbreaks registered in this system were caused by pathogens of the gastrointestinal tract, yet only for a minor portion was reliable evidence available linking these outbreaks to food. This reminds us that outbreaks caused by gastroenteric pathogens, particularly those caused by norovirus, should not be overinterpreted as foodborne outbreaks.

Our data suggest that only 11.3% of reported exposures in outbreaks with >10 cases were statistically significant and associated with the outbreak through case-control or cohort studies. In 37% of the reported foodborne outbreaks in SurvNet, the reporting local health departments were able to associate a meal but not a specific food item with the outbreak. Similarly, Jones et al. have observed that most foodborne outbreak investigations in the United States did not identify a specific food item (*10*). Local health departments must be motivated to improve outbreak investigations to increase the validity of the information received through this system (*29*). In addition to intensifying training programs for the local public health service, RKI is currently developing support tools, such as predesigned electronic line lists of cases and decision-supporting algorithms, to be included in the SurvNet system. Additional training and support tools will assist local health department personnel in the use of epidemiologic methods for outbreak investigations. A new information technology structure will facilitate these additions and further improve the timeliness of the system.

SurvNet has the advantage of being able to document complex multistate outbreaks of any cause. For example, SurvNet was able to capture an outbreak of 1,024 cases of epidemic conjunctivitis, which started within the German armed forces and spread to the civilian population throughout the country (*30*). Comparatively few of the reported outbreaks (3%) were linked to nonnotifiable pathogens or could not be linked to specific pathogens at all. However, this demonstrates that SurvNet is able to cover outbreaks caused by unknown or emerging infectious diseases.

Outbreak surveillance of SurvNet has already provided valuable information for topics of public health relevance. By confirming and quantifying the increase of hospital-based norovirus outbreaks in recent years, SurvNet has contributed to the development of specific recommendations on how to prevent and control norovirus outbreaks in hospitals and nursing homes (*31**–**33*). In 2006, a sharp increase of norovirus outbreak reports was noted at RKI from reporting weeks 43 through 47. This led to a countrywide alert in the national weekly epidemiologic bulletin in week 48 and was subsequently echoed by an alert throughout Europe in the Eurosurveillance Weekly Journal 2 weeks later (*34**–**35*).

Although our report cannot replace a surveillance system evaluation, some system attributes can be addressed. Over a period of 5 years, SurvNet has demonstrated the ability to collect and analyze a large number of outbreak reports in a federal administrative environment of 431 local health departments and 16 federal states in Germany with a total population of 82 million inhabitants. This fact already indicates that requirements of simplicity, acceptability, and stability appear to have been met. SurvNet also seems to compare favorably to other systems in timeliness and sensitivity. The ability of SurvNet to capture outbreaks with unidentified or new pathogens in a systematic way indicates its suitability for outbreak surveillance of emerging infectious diseases. Given the federal structure in Germany and its reflection in the SurvNet design, this system might also be a blueprint for other large national or international outbreak surveillance systems, particularly in the context of the new international health regulations.

## Supplementary Material

Appendix Figure Screen shot of outbreak report in SurvNet. 1) List of smaller outbreaks forming part of the meta outbreak; 2) number of cases in each outbreak; 3) geographic setting; 4) evidence categories by which a food product (here meat) was found to be associated with the outbreak (here by detection of identical pathogen in food and patient); and 5) additional description of outbreak.
