# Supplementary figures and images for: SurvNet Electronic Surveillance System for Infectious Disease Outbreaks, Germany
Source: Emerg Infect Dis. 2007 Oct;13(10):1548–55. doi: 10.3201/eid1310.070253 (PMC2851509; doi:10.3201/eid1310.070253)

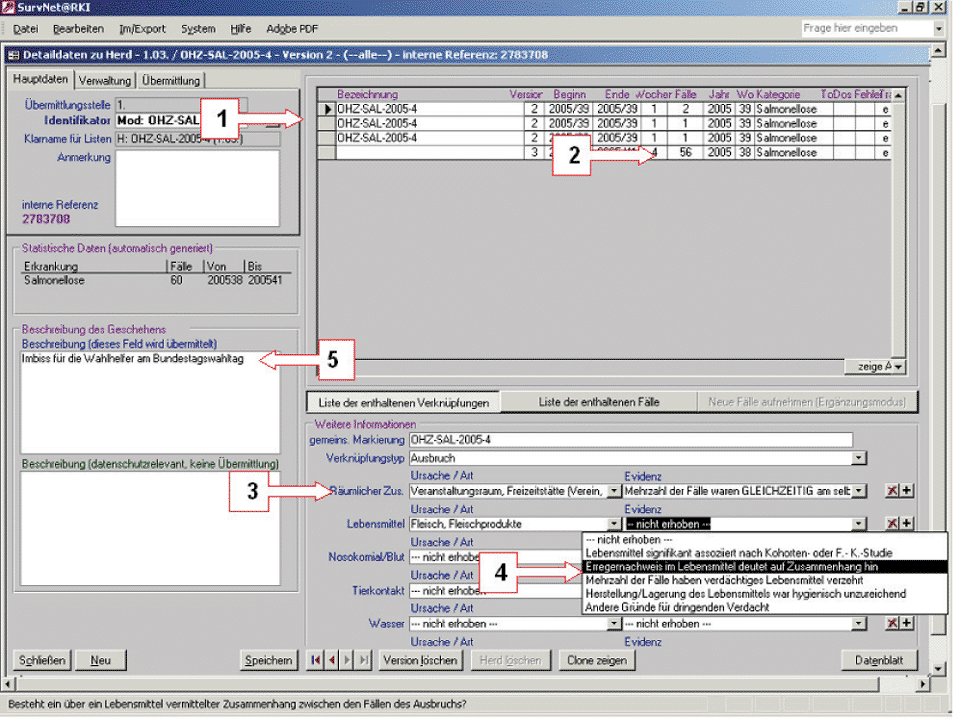

Supplement: Appendix Figure — Screen shot of outbreak report in SurvNet. 1) List of smaller outbreaks forming part of the meta outbreak; 2) number of cases in each outbreak; 3) geographic setting; 4) evidence categories by which a food product (here meat) was found to be associated with the outbreak (here by detection of identical pathogen in food and patient); and 5) additional description of outbreak. [file 07-0253_appF-s1.gif]
